# Supplementary material for: Oligodendrocytes depend on MCL-1 to prevent spontaneous apoptosis and white matter degeneration
Source: Cell Death Dis. 2021 Dec 6;12(12):1133. doi: 10.1038/s41419-021-04422-z (PMC8648801; doi:10.1038/s41419-021-04422-z)
Supplement: Supplementary file 1 — Supplementary Figures [file 41419_2021_4422_MOESM1_ESM.pdf]

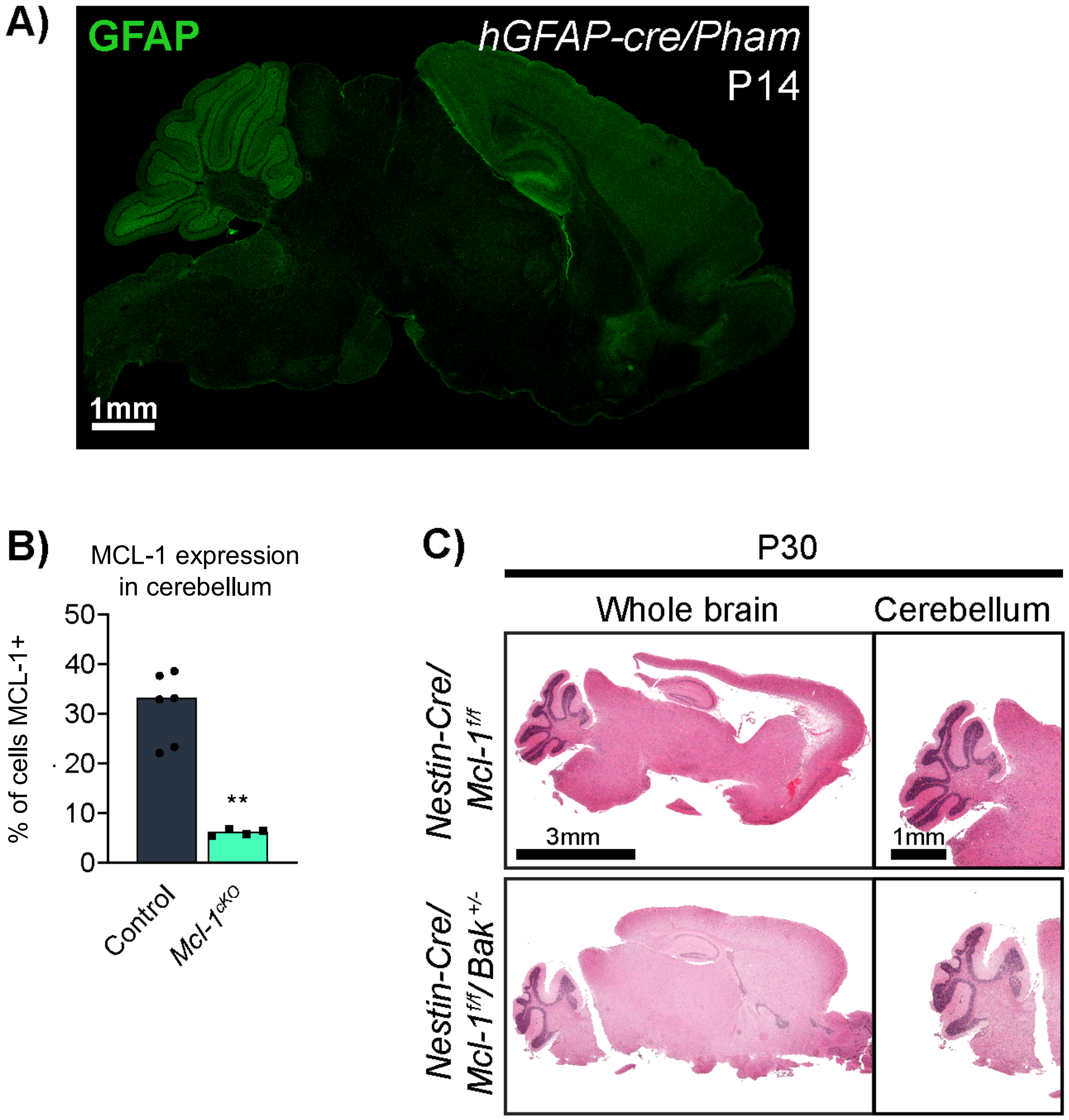

Supplementary Figure 1. *hGFAP-Cre* conditionally deletes *Mcl-1* in regions throughout the brain and conditional *Mcl*-1 deletion driven by *Nestin-Cre* produces a similar phenotype. (A) *hGFAP-Cre* induces expression of the fluorescent reporter DENDRA2 in *hGFAP-cre/Pham* mice, indicating regions of *hGFAP-Cre* expression. *hGFAP-Cre* targets cells in diverse regions throughout the brain, including the cerebellum, hippocampus, and cortex. **(B)** Flow cytometry using MCL-1 immunofluorescence shows reduced MCL1+ cells in dissociated cerebella of *Mcl-1*^{cKO} mice. **(C)** Like *Mcl-1*^{cKO} mice, *Nestin-Cre/Mcl-1*$^{loxP/loxP}$ mice showed white matter degeneration that was rescued by heterozygous *Bak* co- deletion.

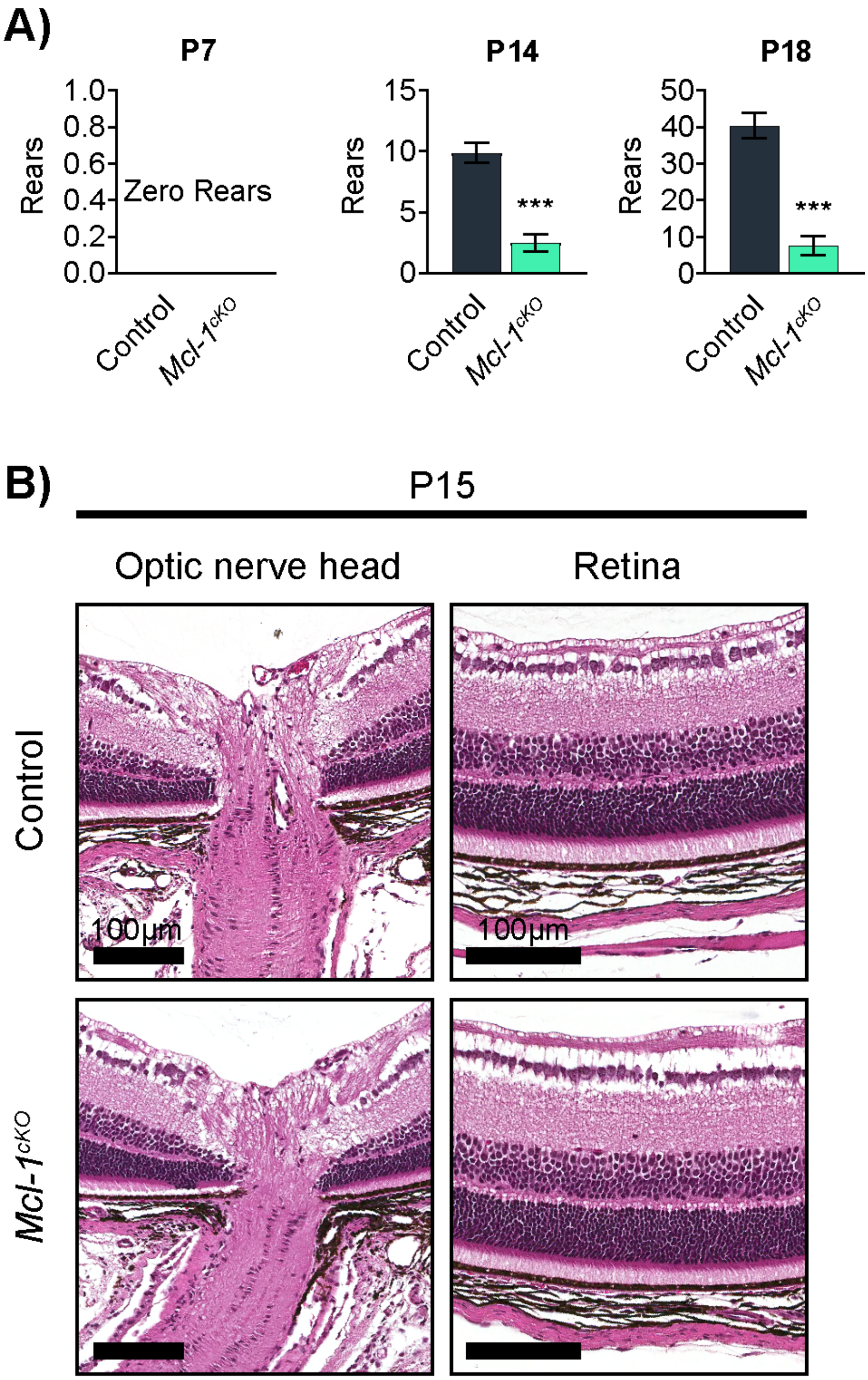

Supplementary Figure 2. Open field rearing behavior changes and absence of retinal pathology in Mcl-1cKO mice. (A) Decreases rearing in *Mcl-1*^{cKO} mice at P14 and P21. (**B)** Normal-appearing retina in *Mcl-1*^{cKO} mice at P15, when brain pathology is fulminant. Scale bar = 0.1mm.

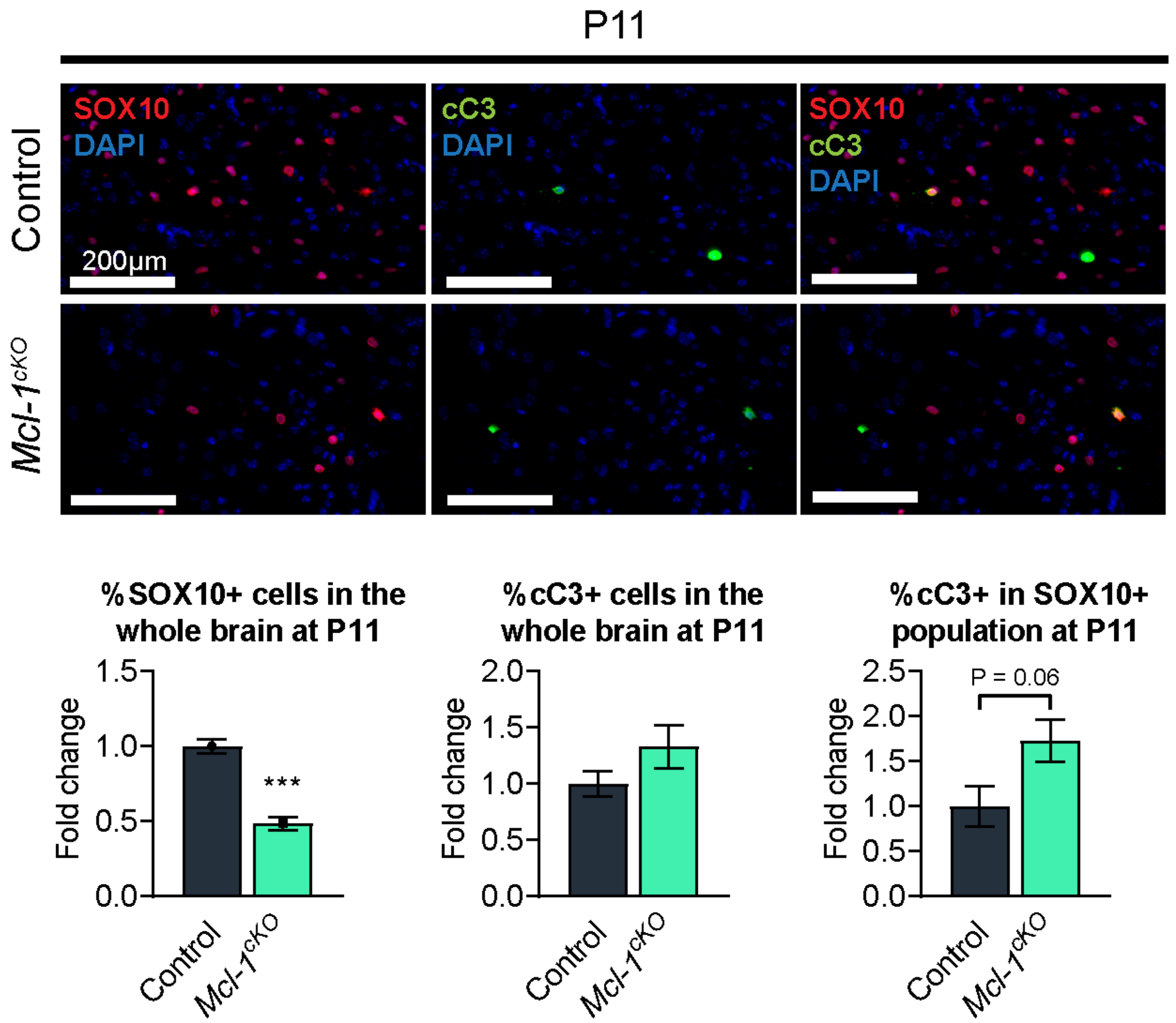

Supplementary Figure 3. Immunofluorescence for cC3 and SOX10 in *Mcl-1^{cKO}* mice and controls at P11. SOX10 is significantly decreased, but no statistically significant changes are noted in the total cC3+ cells or cC3+ cells within the SOX10+ subset.
